# Persistent cutaneous phaeohyphomycosis caused by *Brunneofusispora hyalina* in a patient with a heterozygous *CARD9* variant: the opportunistic potential of a newly described environmental fungus

**DOI:** 10.3389/ffunb.2026.1938341

**Published:** 2026-10-13

**Authors:** Huan Mei, Dongmei Li, Danrui Jing, Xincun Wang, Shuwen Deng, Paul Daly, Hailin Zheng, Xiaodong She, Meihua Fu, Weida Liu

**Affiliations:** 1 Department of Medical Mycology, Hospital for Skin Diseases, Institute of Dermatology, Chinese Academy of Medical Sciences and Peking Union Medical College, Nanjing, China; 2 Chinese Academy of Medical Sciences Collection Centre of Pathogen Microorganisms-D (CAMS-CCPM-D), Nanjing, China; 3 Department of Microbiology & Immunology, Georgetown University Medical Center, Washington, DC, United States; 4 Dermatology and Venereology, Sichuan Academy of Medical Sciences and Sichuan Provincial People's Hospital, Chengdu, China; 5 State Key Laboratory of Microbial Diversity and Innovative Utilization, Institute of Microbiology, Chinese Academy of Sciences, Beijing, China; 6 Department of Medical Microbiology, The People’s Hospital of Suzhou New District, Suzhou, China; 7 Institute of Plant Protection, Jiangsu Academy of Agricultural Sciences, Nanjing, China

**Keywords:** antifungal susceptibility, Brunneofusispora hyalina, Card9 knockout, dematiaceous fungi, murine model

## Abstract

**Objectives:**

This report documents the first isolation of rare dematiaceous fungus *Brunneofusispora hyalina* from a cutaneous lesion in a patient with heterozygous *CARD9* variant. The study characterizes this novel pathogen and simulates infection and treatment processes to understand this opportunist and explore therapeutic strategies.

**Methods:**

Direct microscopy and histopathology confirmed the fungal infection. By collecting morphological images of the isolate, assessing temperature-dependent growth, performing multi-locus and whole-genome sequencing, and conducting antifungal susceptibility testing, the new pathogen’s virulence and antifungal resistance were comprehensively analyzed. Using CARD9-deficient mice to establish infection models and administer drug treatments.

**Results:**

The patient, confirmed by whole-exome sequencing to have a heterozygous *CARD9* variant, was diagnosed with phaeohyphomycosis caused by *B. hyalina* through barcode sequencing. Meanwhile, functional annotation revealed metabolic versatility and enrichment in secondary metabolite pathways, while 395 PHI homologs were identified mainly from plant-pathogenic fungi. The strain exhibited abundant hyphal anastomosis and psychrotolerant growth. Antifungal susceptibility tests using modified agar dilution and broth dilution methods showed low MICs to voriconazole, posaconazole, itraconazole, and terbinafine, with values of 0.5-1 µg/mL, 0.5–2 µg/mL, 1–2 µg/mL, and 0.25–0.5 µg/mL, respectively. However, fluconazole showed a high MIC (128 µg/mL). In contrast, amphotericin B inhibitory concentrations differed substantially between two testing methods, with discrepancies exceeding three twofold dilution titers. In a *CARD9* knockout mouse model, *B. hyalina* caused persistent inflammatory infections without mortality, whereas infection in wild-type mice had self-limiting symptoms. Despite six weeks of combined antifungal therapy, complete eradication of infection was not achieved.

**Conclusions:**

*B. hyalina* is a new opportunistic fungus causing phaeohyphomycosis, particularly in individuals with *CARD9* variants. Genomic analysis shows that *B. hyalina* shares many genes with plant pathogens and lacks specialized human virulence factors, suggesting that host immune vulnerability is key to infection. While antifungal drugs such as triazoles and terbinafine demonstrate low inhibitory concentrations *in vitro*, combination therapy in mouse models reduces fungal burden but does not completely clear the infection, indicating the necessity for prolonged treatment in immunocompromised hosts. Future exploration may focus on immune restoration and hyperthermia, which may complement antifungal therapy and improve outcomes.

## 1 Introduction

Cutaneous phaeohyphomycosis is an uncommon fungal infection caused by melanized (dematiaceous) fungi that are widely distributed in soil, wood, and decaying plant material. Histopathologically, phaeohyphomycosis is distinguished from chromoblastomycosis by the absence of muriform (sclerotic) bodies and is characterized by the presence of brown to black yeast-like cells, pseudohyphae, or septate hyphae within infected tissue. Disease is caused by a diverse group of dematiaceous fungi, including *Alternaria* spp., *Bipolaris* spp., *Cladophialophora* spp., *Cladosporium* spp., *Exophiala* spp., and *Phialophora* spp. among many others (Revankar and Sutton, 2010; He et al., 2022). Most of these fungi are saprophytes and many are often recognized as plant pathogens with a worldwide distribution (Manamgoda et al., 2014; Ha et al., 2015). Although they rarely cause disease in healthy individuals, immunocompromised patients or those receiving immunosuppressive therapy are particularly susceptible to opportunistic infections that may progress from localized cutaneous lesions to invasive diseases (Revankar and Sutton, 2010; Latawa et al., 2022).

Here, we report the first case of cutaneous phaeohyphomycosis caused by *Brunneofusispora hyalina*, a member of the order *Pleosporales*, in a Chinese woman. The infection initially developed on the cheek following local trauma and gradually extended to the thigh over a thirty-year period. The patient, who resided in Xuzhou, a city located north of the Yangtze River, presented with a chronic dark purplish-red plaque with crusting on the cheek that had recently progressed and disseminated. Before the present investigation, the etiological diagnosis remained unresolved, and prolonged antifungal therapy failed to achieve sustained clinical improvement. Notably, the lesion exhibited marked seasonal variation, with improvement during the summer and exacerbation during the winter, suggesting an association with the fungus’s psychrotolerant growth characteristics. Histopathological examination and direct microscopic analysis of biopsy specimens revealed pigmented septate hyphae, consistent with phaeohyphomycosis.

*Brunneofusispora hyalina* is a recently described species within the order *Pleosporales* and has previously been reported only once, as a saprotrophic fungus isolated from decaying wood submerged in a freshwater stream (Mark et al., 2021). To our knowledge, this represents the first report of *Brunneofusispora* spp. causing opportunistic human infection. Further clinical investigation revealed that the patient carried two heterozygous variants in the Caspase Recruitment Domain-containing protein 9 (*CARD9*) gene. Previous studies have suggested that variants in the *CARD9* gene result in an inborn error of antifungal immunity, impairing interleukin-17 (IL-17)-mediated responses and predisposing affected individuals to invasive fungal infection (Wang et al., 2014).

In this study, we comprehensively characterize this emerging opportunistic pathogen through multi-locus identification, morphological and physiological analyses, antifungal susceptibility testing, and whole-genome sequencing to investigate genomic features potentially associated with pathogenicity. Additionally, we established a *B. hyalina* infection model in *CARD9* knockout (KO) mice to evaluate disease progression, host susceptibility, and therapeutic responses. Together, these findings provide new insights into the pathogenic potential of this previously unrecognized fungus and the critical role of CARD9-mediated immunity in preventing opportunistic phaeohyphomycosis.

## 2 Materials and methods

### 2.1 Experimental animals

C57BL/6 wild-type (WT) mice used in this study were purchased from Jiangsu Huachuang Sino Pharma Tech Co., Ltd. *CARD9* knockout (KO) mice on the C57BL/6 background were generously provided by Professor Xinming Jia, Tongji University School of Medicine, Shanghai, China. All experimental procedures used male mice aged 6–8 weeks exclusively. The mice were maintained in a specific pathogen-free facility at ClinBridge Biotech Co. Ltd. Because of the small sample size and exploratory nature of this study, data were organized in Microsoft Excel 2021 and are presented descriptively as mean ± SD. The entire animal experimentation process was conducted strictly in accordance with institutional guidelines.

### 2.2 Isolation and mycology

Two dermal biopsy specimens were obtained from the patient. One specimen was inoculated onto Sabouraud Glucose Agar and incubated at 26 °C for a duration of two weeks, while the other specimen underwent direct microscopic examination.

The isolated fungal strain was subsequently cultured on various media, including Potato Dextrose Agar (PDA), Oat Meal Agar (OA), Malt Extract Agar (MEA), Potato-Carrot Agar (PCA), Synthetic Low Nutrient Agar (SNA), and 1% Tween 80-Corn Meal Agar (CMA), and incubated at 26 °C for 14 days to examine the development of characteristic morphological features. Slide cultures were also prepared on the aforementioned medium to assess sporulation and other microscopic characteristics. These cultures were incubated at 26 °C for up to 28 days under varying light conditions, including darkness, daylight, and near-ultraviolet light.

Furthermore, to assess the influence of temperature on fungal growth, the isolate was inoculated onto PDA plates and incubated at seven different temperatures ranging from 4 °C to 40 °C. Colony diameters were measured over a 28-day period to evaluate growth responses across this temperature gradient.

### 2.3 DNA extraction, PCR amplification and sequencing

For the extraction of genomic DNA, the fungal isolate was cultivated on PDA at 26 °C in darkness for a period of 14 days. Mycelial samples weighing between 50 and 60 mg were collected from the actively growing margins of the cultures. The harvested mycelium was disrupted in tubes containing 1 mm silica beads (MP Biomedicals™, Lysing Matrix C, India) using the FastPrep-24™ 5G homogenizer (Fisher Scientific Inc., Waltham, MA, USA) for 20 seconds. DNA extraction was subsequently performed utilizing the HP Fungal DNA Kit (Omega Bio-tek, Inc., Norcross, GA, USA) following the manufacturer’s protocol.

Polymerase chain reaction (PCR) amplification was performed to amplify the internal transcribed spacer (ITS), large subunit (LSU), translation elongation factor 1-alpha (TEF1-α), and the second largest subunit of RNA polymerase II (RPB2) gene regions using the respective primer pairs. Each PCR was carried out in a total reaction volume of 25 μL, comprising 10 ng of genomic DNA, 12.5 μL of Hot Start High-Fidelity 2X Master Mix (New England BioLabs, Ipswich, MA, USA), and 0.5 μL of each 10 mM primer. Sequencing was conducted by Biozeron Biotechnology Company (Shanghai, China), and the resulting sequences were edited and assembled using BioEdit version 7.2 (Hall, 1999). Multiple sequence alignments were performed and manually refined using MEGA version 7 (Kumar et al., 2016). To elucidate the phylogenetic relationships among the taxa, a Neighbor-Joining (NJ) phylogenetic tree was constructed, employing *Alternaria infectoria* CBS 210.86 as the outgroup.

### 2.4 Genome sequencing, assembly and gene prediction

Whole-genome sequencing of the isolate was conducted utilizing the Illumina NovaSeq 6000 platform with PE150(2×150)reads(Shanghai Biozeron Biotechnology Co., Ltd., Shanghai, China). Library information used the TruSeq™ Nano DNA Sample Prep Kit reagents and methodology. Raw sequencing reads were generated through the Illumina base calling software CASAVA v1.8.2 (http://support.illumina.com/sequencing/sequencing_software/casava.ilmn), following the protocols described in the corresponding documentation. Contaminant reads, including those containing adapter or primer sequences, were identified and removed using Trimmomatic (http://www.usadellab.org/cms/uploads/supplementary/Trimmomatic) with default settings. The resulting high-quality clean reads were subsequently employed for downstream analyses.

Genome assembly was performed using ABySS (http://www.bcgsc.ca/platform/bioinfo/software/abyss) with multiple k-mer parameters to optimize assembly quality. To address residual local gaps and correct single nucleotide polymorphisms, GapCloser (https://sourceforge.net/projects/soapdenovo2/files/GapCloser/) was applied to the assembled contigs. Repetitive elements within the genome were identified using RepeatMasker (http://www.repeatmasker.org/) under default parameters.

For gene prediction, gene models were delineated using Augustus v3.2.3. Functional annotation of predicted proteins was conducted by performing BLASTp searches against several databases, including the NCBI non-redundant protein database (NR), SwissProt (http://uniprot.org), Kyoto Encyclopedia of Genes and Genomes (KEGG, http://www.genome.jp/kegg/), and Clusters of Orthologous Groups of proteins (COG, http://www.ncbi.nlm.nih.gov/COG). Additionally, transfer RNA (tRNA) genes were identified using tRNAscan-SE v2.04 (http://lowelab.ucsc.edu/tRNAscan-SE), and ribosomal RNA (rRNA) genes were detected with RNAmmer v1.2 (http://www.cbs.dtu.dk/services/RNAmmer/).

### 2.5 Antifungal susceptibility test

Because the isolate failed to produce conidia under the culture condition described in section 2.1 and exhibited relatively slow growth, the standard Clinical and Laboratory Standards Institute (CLSI) M38 broth microdilution method, which relies on a standardized conidial inoculum, was not directly applicable. Therefore, antifungal susceptibility was evaluated using both a modified agar dilution test (m-ADT) (Yoshida et al., 1997) and a modified broth dilution test (BDT) adapted from the CLSI M38 guidelines. The isolate was tested against fluconazole, itraconazole, voriconazole, posaconazole, amphotericin B, and terbinafine. The concentration ranges for all antifungals were from 0.016 to 16 μg/mL, except for fluconazole, which was tested over a range of 0.13 to 256 μg/mL. All antifungal compounds were obtained from Sigma-Aldrich.

Preparation of the antifungal agents involved accurately weighing predetermined amounts of each compound, which were subsequently dissolved in dimethyl sulfoxide (DMSO; Sangon Biotech, Shanghai, China) to achieve the desired stock concentrations. For m-ADT, serial twofold dilutions of each drug were then prepared in RPMI-1640 medium supplemented with 2% glucose and 2% agar, buffered with 3-(N-morpholino) propanesulfonic acid (MOPS) at pH 7.0 (Guinea et al., 2019). Fresh fungal inoculation, consisting of 2 mm square blocks, were aseptically placed at the center of the agar plates containing the respective antifungal concentrations, as well as on control plates lacking antifungal agents. The inoculated plates were incubated at 26 °C for seven days, after which the diameters of fungal colonies were measured. The Minimum Inhibitory Concentration (MIC) was defined as the lowest antifungal concentration at which the colony diameter did not exceed 2 mm; During inoculation, use a tissue lyser set at 2,500 cpm with shaking for 30 seconds. Next, let the mixture settle, then filter it and count the cells under a microscope (each cell with an intact cross-wall is considered a single cell). Prepare a working suspension with 3 × 10^4^ hyphal fragments per milliliter. Then, incubate the inoculated 96-well plate containing the drug at 26 °C for 7 days, until strong growth is observed in the positive control wells. Throughout the procedure, ATCC 22019 and ATCC 6258 strains are used as quality controls. All experiments are conducted in triplicate and repeated twice on separate occasions to confirm reproducibility.

### 2.6 Establishment of an infection and treatment model for phaeohyphomycosis in CARD9 KO mice

Using the aforementioned grinding and counting methods, a fungal suspension of *B. hyalina* at a concentration of 2 × 10^8^ hyphal fragments per milliliter was prepared and quantitatively collected. Wild-type (WT) and *CARD9* knockout (KO) mice were randomly assigned to experimental groups. A total of 40 WT mice served as controls and were divided into eight groups to record paw swelling weekly from weeks 1 to 8 post-infection, with five mice assessed each week. Concurrently, 70 *CARD9* KO mice were used: 40 were designated as the infection group, with five mice evaluated weekly from weeks 1 to 8 post-infection to measure paw swelling and to perform tissue grinding and counting after excision of the paw mass. The remaining 30 mice formed the treatment group; from the third week of infection, they began receiving voriconazole (Vfend^®^, Pfizer Italia S.r.l., Ascoli Piceno, Italy) and terbinafine (Lamisil^®^, Beijing Novartis Pharma Co., Ltd., China) treatment, and five mice per week were assessed for paw swelling and excised mass analysis until week 8. Each infected mouse received a subcutaneous inoculation of 50 μL of the fungal suspension into the left hind paw, while the contralateral paw was injected with an equal volume of sterile saline as a negative control (Wu et al., 2016).

To assess antifungal efficacy, *CARD9* knockout mice in the treatment group received oral gavage with a combination of voriconazole and terbinafine in the third week post-infection. The dosages were calculated based on standard adult clinical doses and converted using the body surface area (BSA) normalization method. Clinically, the typical oral adult doses for voriconazole and terbinafine are 200 mg/day and 250 mg/day, respectively. Assuming an adult weight of 80 kg, these doses correspond to 2.50 mg/kg/day for voriconazole and 3.125 mg/kg/day for terbinafine. Using the BSA-based interspecies dose conversion method described by Reagan-Shaw et al. (2008), where the Km factors for adults and mice are 37 and 3, respectively, the equivalent mouse doses for voriconazole and terbinafine were calculated to be approximately 30 mg/kg/day and 40 mg/kg/day. The calculation formula used is as follows:


$$\text{Mouse dosage}(\frac{\text{mg}}{\text{kg}})=\text{human dose}(\frac{\text{mg}}{\text{kg}})\times \frac{\text{Km}(\text{Human})}{\text{Km}(\text{Mouse})}$$

Throughout the experimental period, paw swelling was measured using a micrometer. At specified time points, homogenates of infected paws were serially diluted and plated onto Sabouraud dextrose agar (SDA) to determine fungal burden. Tissue specimens were collected and subjected to hematoxylin and eosin (H&E) staining and periodic acid–Schiff (PAS) staining for histopathological examination to assess tissue inflammation and fungal invasion.

## 3 Results

### 3.1 Case presentation

The clinical specimen was obtained from the cheek of a 35-year-old female patient. Approximately 30 years before presentation, the patient sustained facial trauma associated with exposure to coal and wood, which was subsequently followed by the emergence of small, dark purplish-red papules. Over time, the lesions gradually enlarged, coalesced, and became more deeply pigmented without ulceration. The patient reported mild pain and intermittent pruritus.

A skin biopsy performed approximately twenty years earlier revealed pigmented fungal elements, including thick-walled spores with budding; however, fungal culture failed to establish a definitive etiological diagnosis. Although repeated courses of intravenous and oral antifungal therapy resulted in partial clinical improvement, the lesions persisted with recurrent exacerbation. Notably, the disease showed marked seasonal variation, with worsening during the winter and partial remission during the summer. Upon representation in the winter of 2023, the lesions had progressively extended to involve the ear, neck, and right thigh.

During the examination, multiple direct microscopic analyses of skin specimens, conducted both prior to and during treatment, consistently demonstrated the presence of pigmented septate hyphae. Efforts to identify muriform bodies were specifically undertaken but yielded negative results. Additionally, repeated attempts to detect the thick-walled budding spores described historically were unsuccessful. (**Figure 1A**). The patient experienced a disease recurrence one year after discontinuing treatment, and the same dematiaceous fungus was isolated and identified again. After incubation on PDA medium for two weeks, the isolate produced dark olive-green, velvety colonies (**Figure 1B**). Histopathological analysis revealed abundant pigmented hyphae within the epidermis and dermis, accompanied by granulomatous inflammation in the dermis (**Figure 1C**).

**Figure 1:**
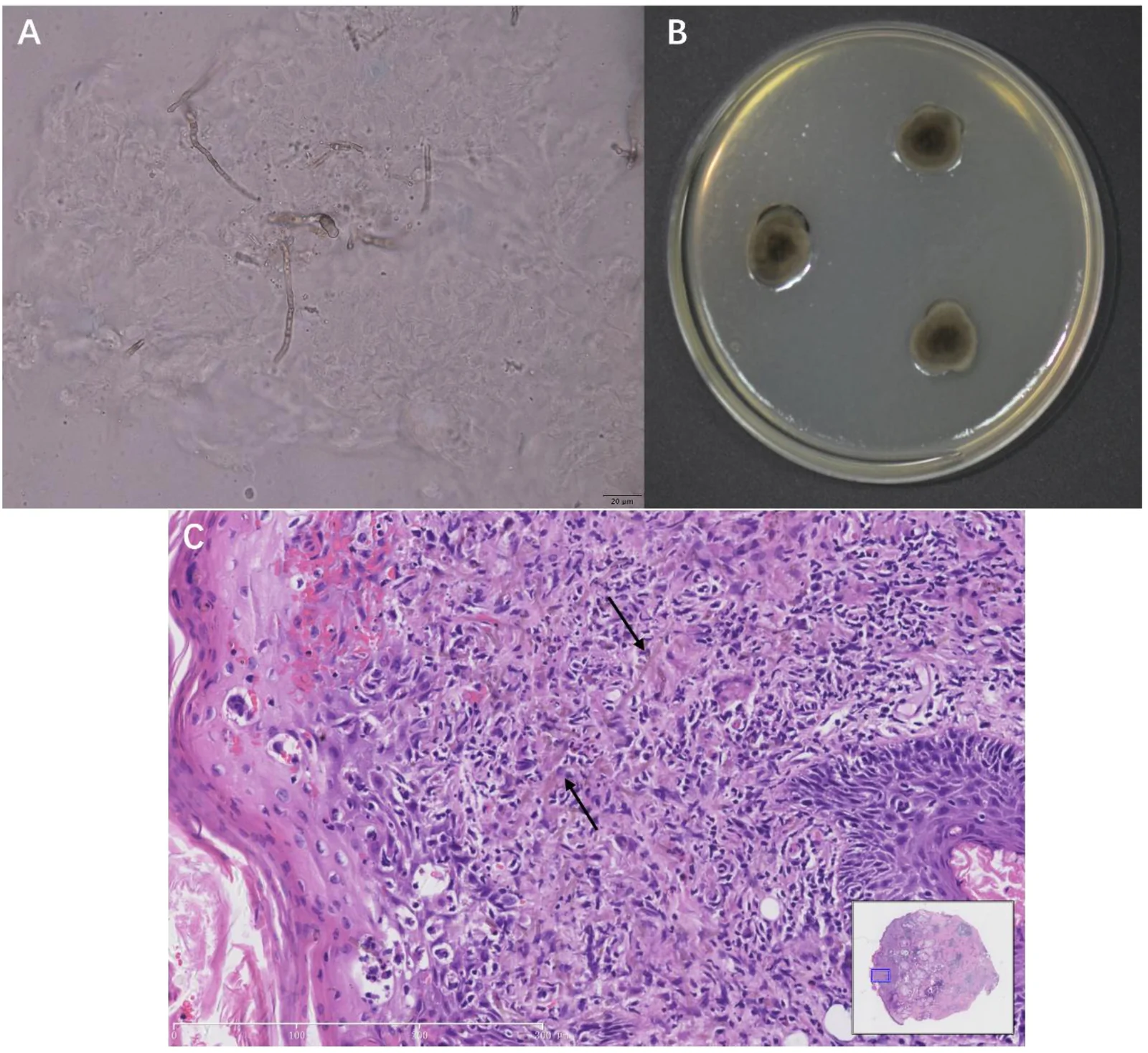
**(A)** Direct microscope examination of cultured fungi revealed septate brown hyphae without sporulation (×400); **(B)** 14-day old isolation on PDA at 26 °C showing deep olive-green velvet colony; **(C)** Histological examination for the infected tissue was taken and PAS staining showed numbers of septate fungal hyphae (black arrow) disseminated throughout the tissue. (×600).

Subsequent whole-exome sequencing of the patient revealed two heterozygous variants of undetermined phase in the *CARD9* gene, including a frameshift variant (transcript NM_052813.5) (c.188_191dup, p.Leu65Alafs*59) and a synonymous variant in exon 3 (c.951G>A, p.Arg317=). The former variant was classified as pathogenic, while the latter was classified as variant of uncertain significance (VUS). Therefore, we suspect that above mutations may increase the patient’s susceptibility to environmental fungi (Nicholas et al., 2016; Wu et al., 2016; Wang et al., 2018).

After the pathogen has been identified, the patient was initially treated with oral posaconazole. On day 1, the dose was 300 mg every 12 hours; from day 2 onward, posaconazole was administered at 300 mg qd for one year. One month after discontinuing treatment, the condition relapsed. Based on antifungal susceptibility testing, terbinafine 250 mg qd was added to the ongoing oral posaconazole 300 mg once daily. The patient has now been on this combination regimen for over a year, with good disease control.

### 3.2 Morphological and physiological characteristics of the strain

After being cultured on various media for 14 days, the isolate developed colonies with the following diameters: MEA (1.3 cm), OA (2.2 cm), PDA (1.5 cm), PCA (1.5 cm), SNA (1.4 cm), and CMA (1.8 cm), respectively. Colonies were velvety with slightly raised centers and olive-grey aerial mycelia. Diffusible brown pigment was observed only on 1% Tween 80-CMA, whereas no pigment diffusion was detected on the other media.

Despite cultivation on multiple sporulation-inducing media for up to 28 days at 26 °C, under different lighting conditions, including continuous darkness, daylight, and near-ultraviolet light, no conidia or other reproductive structures were observed. Instead, slide cultures consistently showed abundant branched, septate brown hyphae measuring 1.8 to 2.5 μm in diameter. The hyphal apices were tapered and less pigmented than the mature hyphae. Extensive hyphal anastomosis was a prominent microscopic feature (**Figure 2**).

**Figure 2:**
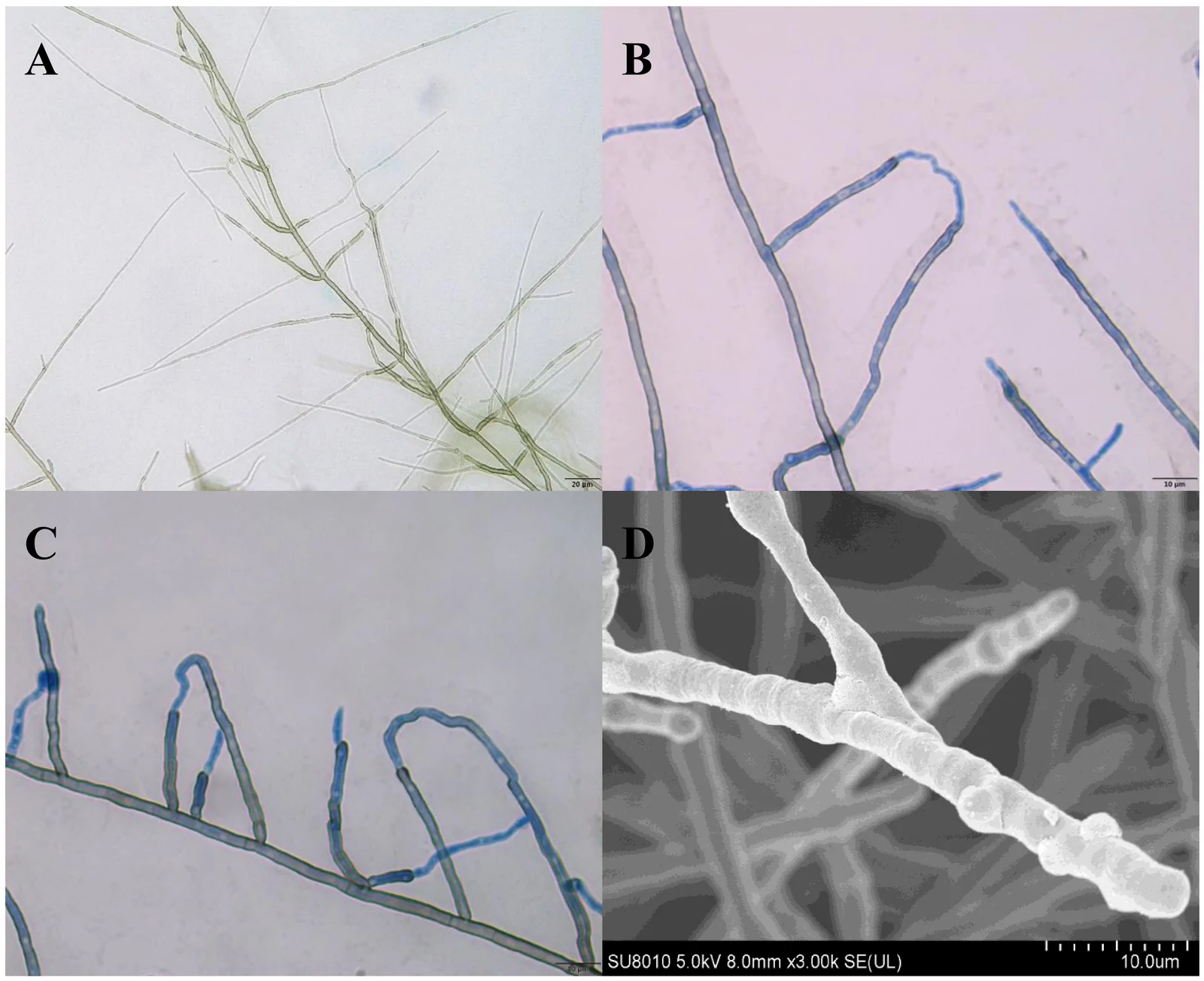
**(A)** After cultivation on PDA for 28 days at 26 °C, microculture showed the brown septate hyphae without sporulation (×400); **(B, C)** To observe the hyphal fusion phenomenon (black arrow) in greater detail, Lactophenol-cotton blue mount was prepared and examined under oil immersion microscopy. The fused hyphal tips are tapered with less melanin deposition, making them more distinctly stained (×1000); **(D)** Under scanning electron microscopy, the hyphae surface appears rough, with noticeable anastomosis occurring between pairs of hyphae (original magnification×4000).

Temperature growth assays demonstrated that the isolate grew over a temperature range of 10–35 °C, with optimal growth at 26 °C (**Figure 3**). Colony expansion remained slow at 10 °C and progressively decreased above 30 °C, with no visible growth at 37 °C or 40 °C. However, cultures pre-incubated at 37 °C resumed growth after being returned to 26 °C, whereas exposure to 40 °C, resulted in irreversible loss of viability, indicating that the upper thermal limit of the isolate lies between 37 °C and 40 °C.

**Figure 3:**
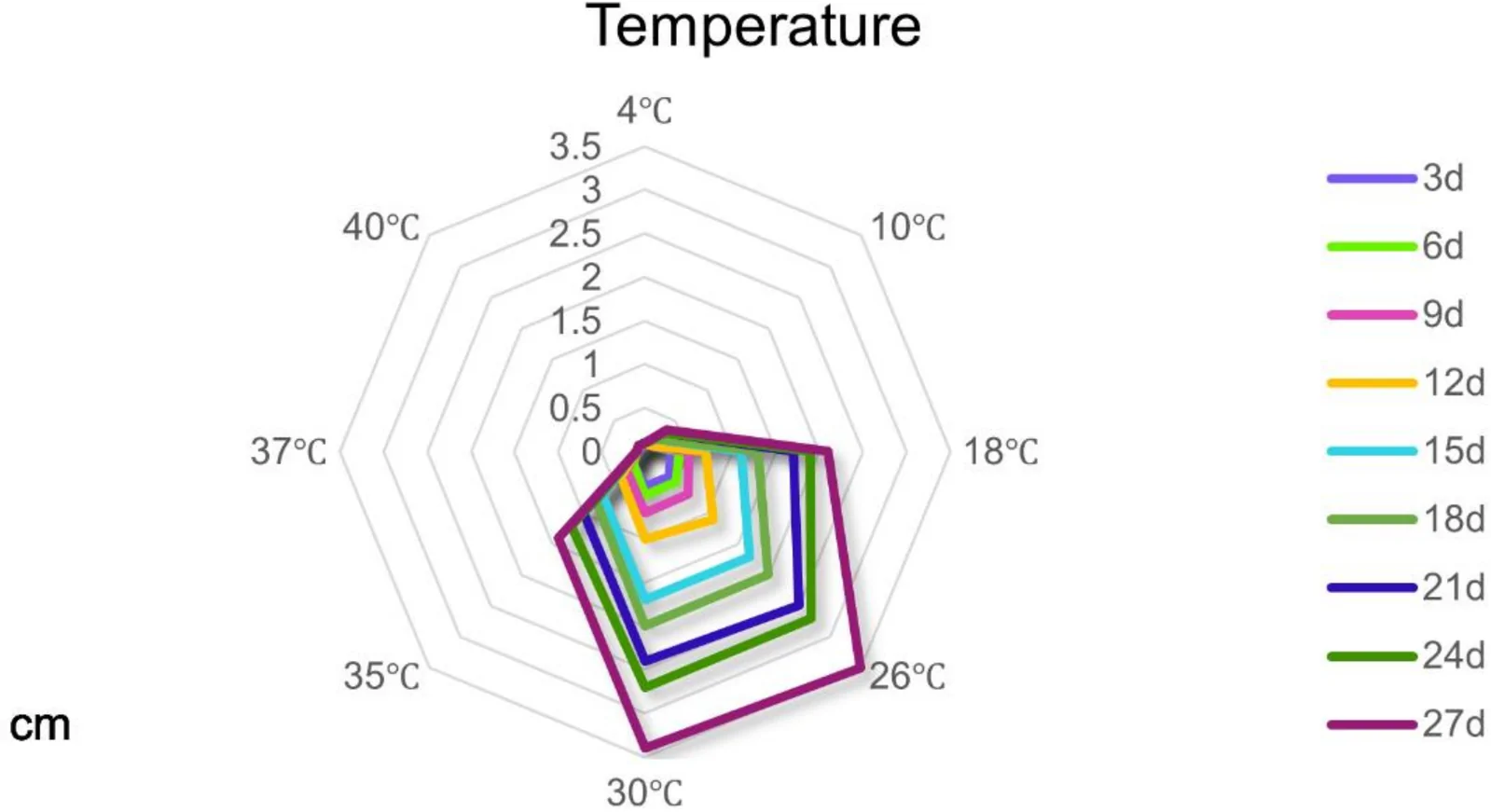
Growth of *B. hyalina* at different temperatures tested on PDA plates.

### 3.3 Molecular identification and phylogenetic analysis of the causative fungus

Because the isolate failed to produce diagnostic reproductive structures (sporulation), molecular identification was performed by amplifying and sequencing four genetic regions, including ITS, LSU, TEF1-α, and RPB2. The resulting sequences were compared with reference sequences in the GenBank using the Basic Local Alignment Search Tool (BLAST) (http://blast.ncbi.nlm.nih.gov/Blast.cgi)?. The highest sequence identity was 98% for ITS (NR_172193.1), 99% for LSU (NG_075405.1), 99% for TEF1-α (MW512606.1), and 98% for RPB2 (MW512609.1), all corresponding to *B. hyalina*. The newly generated sequences were deposited in GenBank under accession numbers LSU(PV643204.1), ITS (PV643203.1), TEF1-α (PV685911.1), and RPB2 (PV685910.1).

Phylogenetic analyses based on the ITS dataset placed the clinical isolate within *B. hyalina* clade, clustering closely with the ex-type strain and providing intuitive support for its taxonomic identification (**Figure 4**).

**Figure 4:**
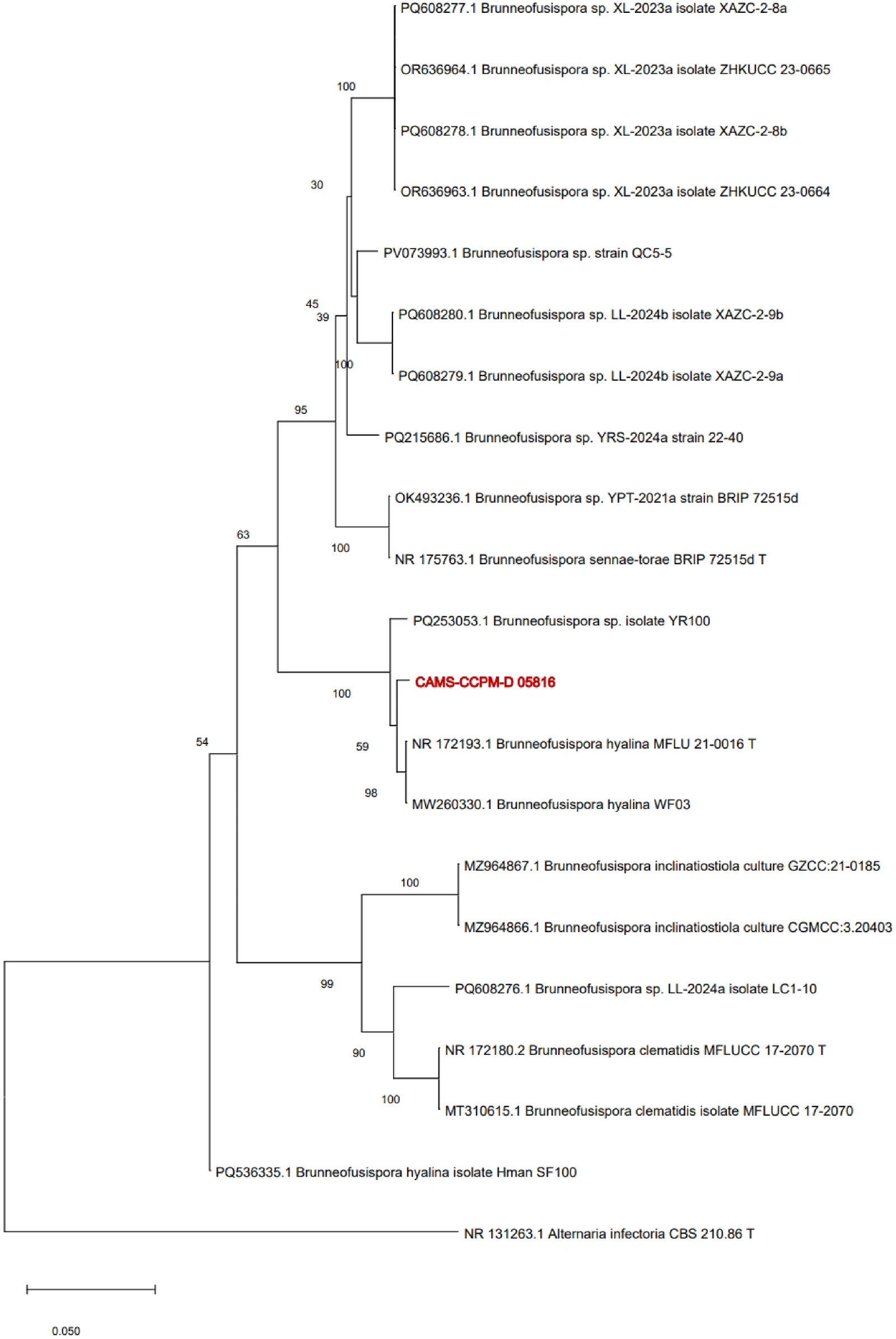
A neighbor-joining (NJ) phylogenetic tree based on *ITS* gene sequences of strain CAMS-CCPM-D 05816.

In summary, we classified this isolation as *B. hyalina* and designated it with the strain number CAMS-CCPM-D 05816.

### 3.4 Whole-genome and gene prediction

The genome of the newly recognized opportunist *B. hyalina* was sequenced using Illumina assembled *de novo*. A total of 5,787 Mb of raw data was obtained, from which 5,603 Mb of high-quality reads were produced. The assembled genome is approximately 44.5 Mb in size, based on 125× sequencing coverage. All raw sequencing data from this study have been submitted to NCBI under BioProject accession number PRJNA1260749, with BioSample accession SAMN48403455 and SRA accession SRR33529083. The genome assembly will be made publicly available upon publication. Genome assembly statistics are summarized in **Table 1**.

**Table 1:** The statistics for the assembled genome.

Assembly statistics	Number
Total scaffolds	219
Total base	44543263
No. of large scaffolds(>1kbp)	190
Bases in large scaffolds	44522552
Largest length	1854493
Scaffold N50	839144
Scaffold N90	245868
G+C content	48.76%
N rate	0%

The assembly genome had a GC content of 48.76%, consistent with values reported for other black yeasts and relatives, such as Chaetothyriales (Ascomycota) (Teixeira et al., 2017). Gene prediction was performed *de novo* using AUGUSTUS version 3.2.3 (http://bioinf.uni-greifswald.de/augustus/), resulting in the prediction of 13,777 genes, with coding regions accounting for 48.3% of the assembled genome Detailed genome annotation statistics are presented in **Table 2**.

**Table 2:** The statistics of gene information.

Gene information	Number/percent
Gene number	13777
Gene total length	21497880
Gene average length	1560
Gene density genes per kb	0.309
GC content in gene region (%)	51.2%
Gene/Genome (%)	48.3%

To functionally annotate the predicted proteins, the Eukaryotic Clusters of Orthologs (KOG) database was used to classify proteins based on sequence homology. A total of 7,912 protein-coding genes were assigned to 26 functional categories (**Figure 5A**). Excluding category S (function unknown), the three most abundant KOG groups were Q, secondary metabolites biosynthesis, transport, and catabolism (930 genes); G, carbohydrate transport and metabolism (547 genes); and E, amino acid transport and metabolism (552 genes), indicating substantial metabolic versatility. Functional annotation against the KEGG database further assigned the predicted genes to diverse biological pathways. As shown in **Figure 5B**, the top three KEGG-annotated pathways were Metabolic pathways, Biosynthesis of secondary metabolites and Microbial metabolism in diverse environments. These extensive metabolic capabilities and a strong potential for secondary metabolite processes are consistent with an environmental saprotrophic lifestyle while also suggesting the presence of metabolic traits that may facilitate opportunistic adaptation in the human host. To identify proteins with potential relevance to virulence or pathogenicity, the predicted proteome was searched against the Pathogen–Host Interactions (PHI) database using BLASTp (E-value ≤ 1e^−5^, ≥70% amino acid identity over ≥70% of the query sequence length). A total of 395 homologs of PHI-annotated proteins were identified, with matches distributed across 38 fungal species. The top five PHI homologs were identified as entries from *Magnaporthe oryzae* (89), which is linked to plant diseases including blast (rice), blast, blast disease (rice); *Fusarium graminearum* (89), associated with plant diseases including fusarium ear blight, fusarium head blight, ear rot in corn, and yellow mold in peanuts; *Aspergillus fumigatus* (55), related to pulmonary aspergillosis and aspergillosis; *Botrytis cinerea* (23)*, connected to plant diseases of grey mold and infection; *Parastagonospora nodorum* (23), implicated in wheat diseases including stagonospora nodorum blotch and glume blotch. Notably, most PHI homologs matched entries derived from plant pathogenic fungi, whereas only a small proportion corresponded to human fungal pathogens, including *A. fumigatus* (12.66%) and *Candida* spp. (2.03%). The matched PHI entries indicated that most of the remaining virulence genes are associated with pathogenicity which are related to plant diseases., consistent with *B. hyalina* possessing a gene repertoire related to plant-associated fungi. However, sequence homology to PHI entries does not by itself confirm a virulence function in *B. hyalina*; a subset of these homologs corresponds to deeply conserved proteins with general cellular functions and are therefore not interpreted as lineage-specific virulence determinants. Furthermore, six proteins shared ≥95% amino acid identity with homologs from members of the order Pleosporales or closely related taxa. Detailed information is provided in **Supplementary Tables 1**, **2**.

**Figure 5:**
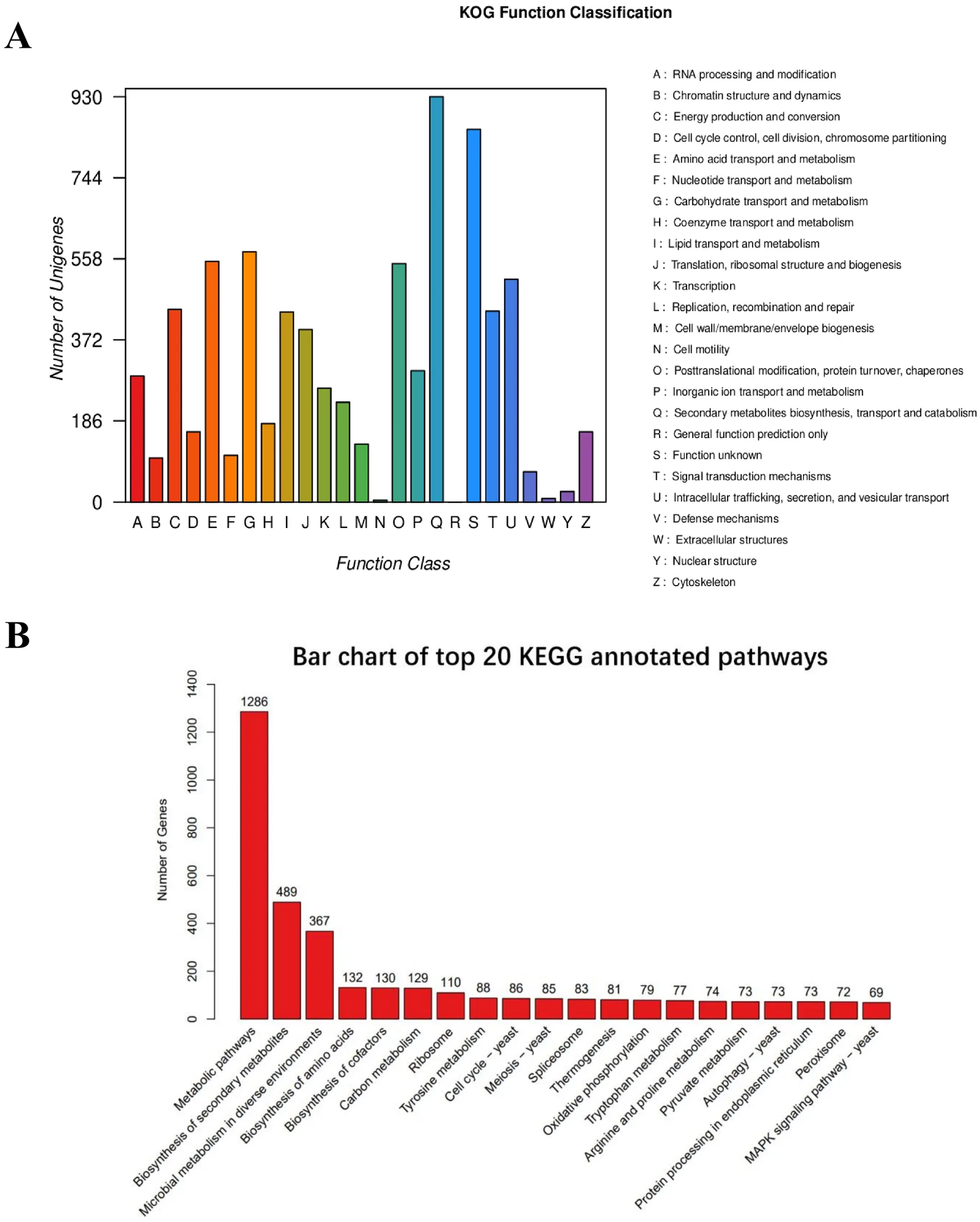
The analysis of Eukaryotic Clusters of Orthologs (KOG) and Kyoto Encyclopedia of Genes and Genomes (KEGG) database. **(A)** KOG class annotation distribution of the *B. hyalina* genome; **(B)** The top twenty KEGG annotated pathways of *B. hyalina* genome.

### 3.5 *In vitro* antifungal susceptibility test

Antifungal susceptibility of *B. hyalina* was evaluated using both the modified agar dilution test (m-ADT) and broth dilution test (BDT), and the minimum inhibitory concentrations (MICs) of six antifungal agents were determined. Using hyphal fragments of *B. hyalina*, the m-ADT assay demonstrated fungistatic activity with MIC values of 1.0 µg/mL for voriconazole and 0.5 µg/mL for terbinafine. In contrast, the isolate exhibited a high MIC value to fluconazole (128 µg/mL). Meanwhile, amphotericin B showed an MIC exceeding 16 µg/mL using the m-ADT method. Similarly, the BDT method revealed a comparable susceptibility profiling. Voriconazole, terbinafine, and posaconazole displayed low MIC values of 0.5 µg/mL, 0.25 µg/mL, and 0.5 µg/mL, respectively, while fluconazole again demonstrated a markedly elevated MIC.

However, a substantial discrepancy was observed for amphotericin B. The MIC determined by the BDT method was 2 µg/mL, representing a difference of more than three twofold dilution steps compared with the value obtained by m-ADT (>16 μg/mL). Detailed susceptibility data are presented in **Table 3**.

**Table 3:** *In vitro* susceptibilities of *B. hyalina* to antifungal agents determined by both modified agar dilution testing method(m-ADT) and broth dilution testing (BDT).

Test Agents	MIC(µg/mL) with m-ADT	MIC(µg/mL) with BDT
Fluconazole	128	128
Posaconazole	2	0.5
Voriconazole	1	0.5
Itraconazole	2	1
Terbinafine	0.5	0.25
Amphotericin B	>16	2

### 3.6 *In vivo* responses to B. hyalina infection and antifungal treatment in CARD9 KO mice

To investigate the role of *CARD9* in host defense against *B. hyalina* and to evaluate therapeutic efficacy based on the *in vitro* susceptibility profiling, WT mice and *CARD9* KO mice were cutaneously inoculated with *B. hyalina*. As illustrated in **Figure 6A**, within the first week following inoculation with *B. hyalina*, WT mice developed acute inflammatory swelling of the injected (left) footpad, with the difference in thickness between the left and right(control) footpads reaching twice that observed in *CARD9* KO mice. However, this inflammation subsided rapidly thereafter. By the second week, the thickness difference between the infected and control footpads in the WT group was already markedly lower than that in the *CARD9* KO group, and by the third week, the difference was less than half of that seen in CARD9-deficient mice. By week 8, the mean difference in thickness between the left and right footpads was only 0.04 mm, indicating that the infection had resolved. In contrast, *CARD9* KO mice developed persistent chronic inflammatory swelling throughout the observation period. The difference in thickness between the infected and control footpads remained between 1.078 and 1.416 mm, with no evidence of spontaneous resolution. Despite the chronic nature of the infection, no mortality was observed in the *CARD9* KO mice during the 8-week study.

**Figure 6:**
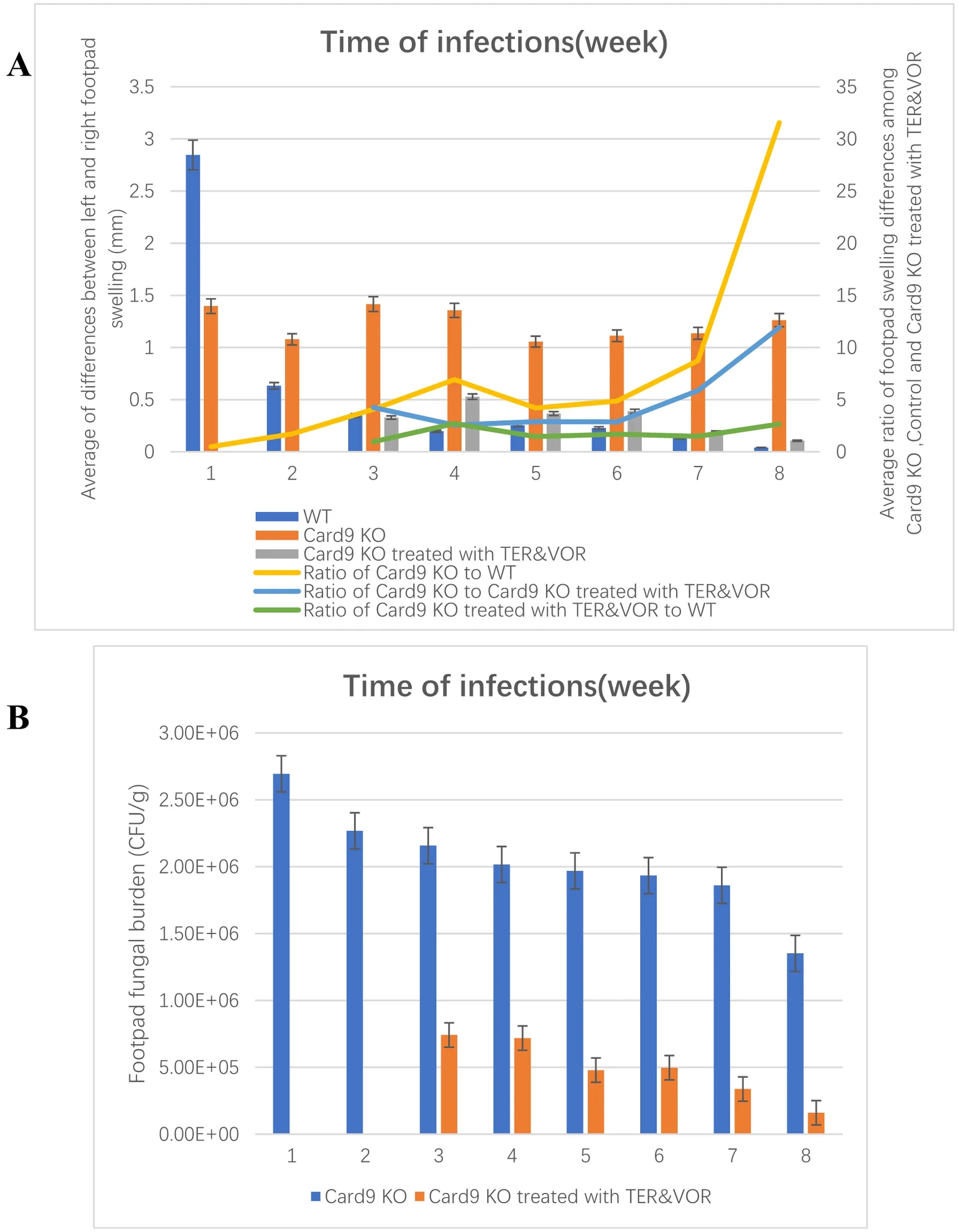
WT and *CARD9* KO mice were infected by inoculating the left footpad with 50 μl of *B. hyalina* suspension (concentration of 2 × 10^8^ hyphal fragments per millilitre), while the right footpad received 50 μl of saline as a control. *Card9* KO mice were divided into treatment and dosing groups and received voriconazole and terbinafine from the third week of post-infection. **(A)** Footpad swelling in WT (n = 5), *CARD9* KO mice (n = 5), and *CARD9* KO mice treated with voriconazole and terbinafine (n = 5) at different time points after infection with *B. hyalina*. Data are presented as mean ± SD of five samples per group; **(B)** Fungal burden in the footpads of *CARD9* KO mice, either treated (n=5) or untreated (n=5), at different time points after infection with *B. hyalina*.

To evaluate antifungal efficacy, infected *CARD9* KO mice received combination therapy with voriconazole and terbinafine beginning in the third week post-infection. During the first week of treatment, the mean paw pad thickness decreased by more than fourfold compared with untreated *CARD9* KO mice. Although inflammation continued to improve throughout the treatment period, residual swelling remained detectable at week 8. The difference in footpad thickness (0.106 mm) relative to WT mice was still more than twice that observed in the WT group (0.04 mm).

Quantitative analysis of fungal burden revealed that, compared with untreated *CARD9* KO mice, the gene-deficient mice receiving co-administration exhibited more than a twofold reduction in fungal load in the infected footpad starting from the first week after treatment (**Figure 6B**). **Figure 7** presents comparative photographs of WT mice and *Card9* KO mice at 4 weeks post-infection, alongside the condition of *Card9* KO mice at the fourth week following post-infection administration of treatment.

**Figure 7:**
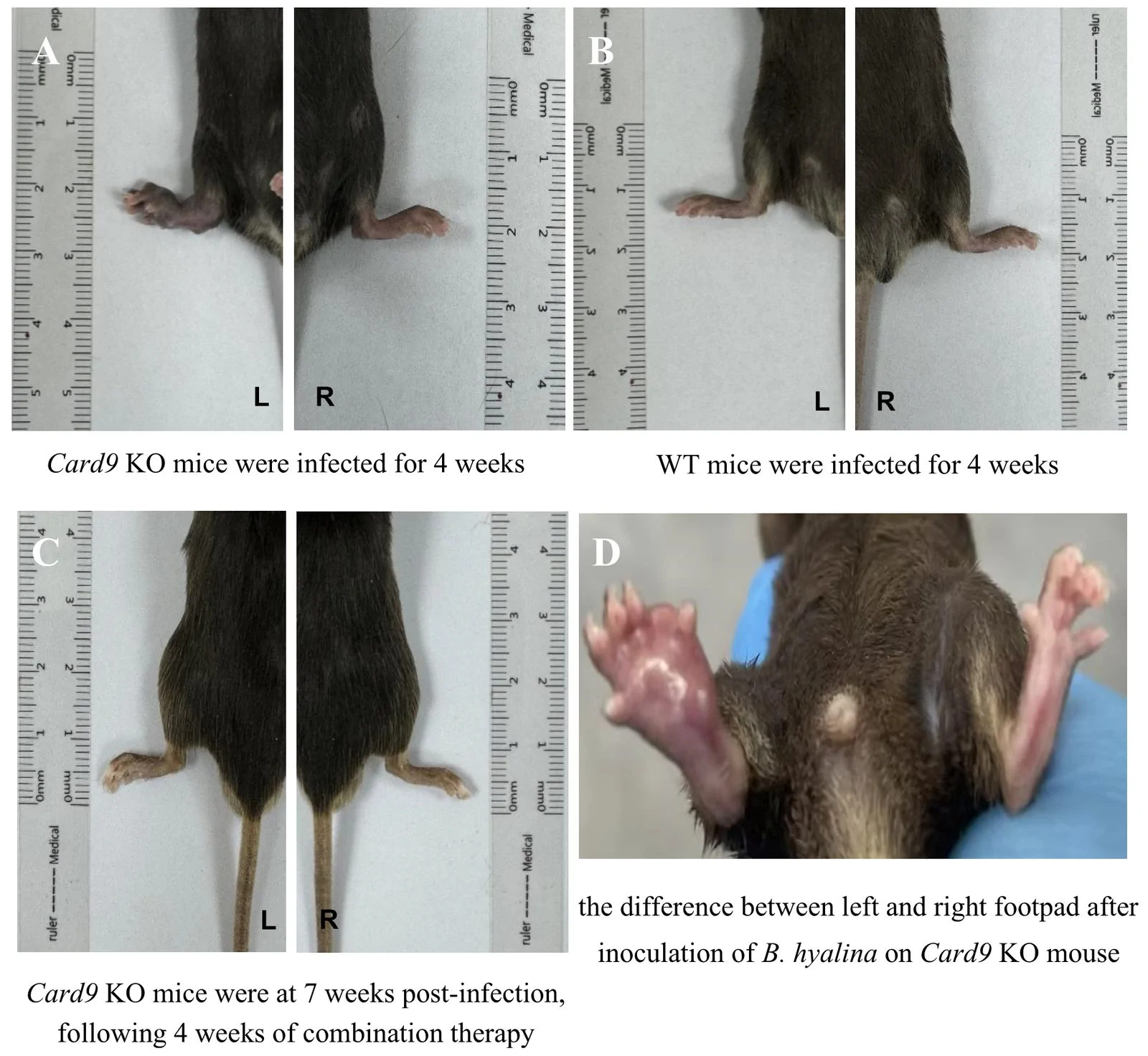
The various phenotypic responses observed in WT mice and *CARD9* KO mice following fungal suspension inoculation and drug administration. **(A)** Comparison of footpad swelling in *CARD9* KO mice four weeks post-infection; **(B)** Comparison of footpad swelling in WT mice four weeks post-infection; **(C)** Comparison of footpad swelling in *Card9* KO mice administered voriconazole and terbinafine at the third week post-infection, after four weeks of combination therapy. **(D)** Differences in foot infection severity in *CARD9* KO mice around the third week.

Histopathological examination using PAS and H&E staining further confirmed the persistent granulomatous inflammation and the presence of residual cutaneous fungal hyphae after six weeks of antifungal therapy (**Figure 8**).

**Figure 8:**
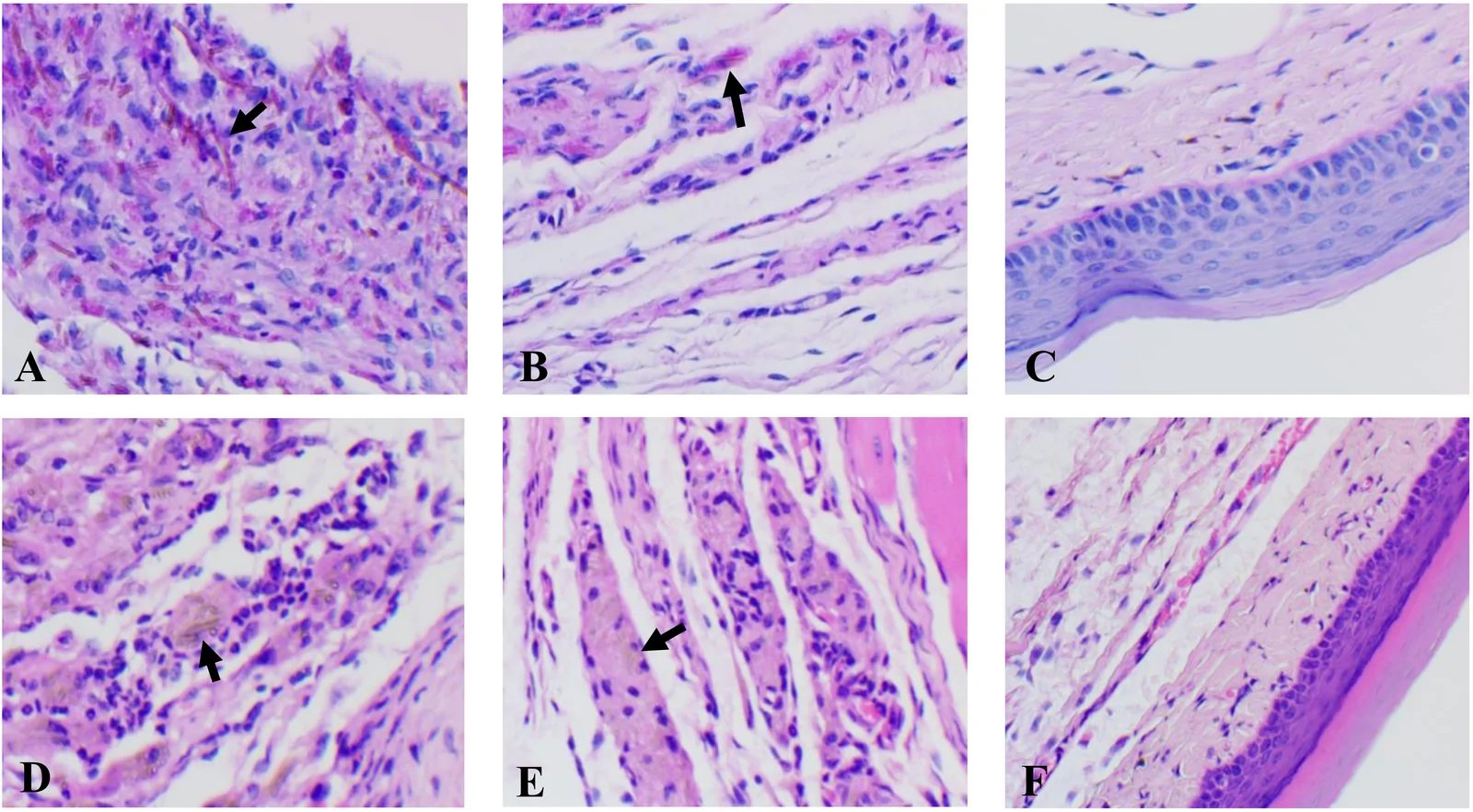
PAS and HE staining results of WT mice and *CARD9* KO mice post- infection, as well as *CARD9* KO mice after treatment. **(A, D)** Infected *CARD9* KO mice exhibited significant thickening of the skin and subcutaneous tissues, inflammatory cell infiltration, and dilated, congested blood vessels; abundant purplish-red (PAS) or brown (HE) fungal hyphae were observed; **(B, E)** After six weeks of treatment, the infected *CARD9* KO mice showed reduced footpad lesions, more orderly arrangement of blood vessels and connective tissues, decreased inflammatory cell infiltration, and near-normal restoration of basement membrane staining. Only seldom fungal hyphae were observed; **(C, F)** WT mice infected for eight weeks displayed intact epidermal structure in the footpad, appropriately thick stratum corneum, regular dermal and subcutaneous connective tissues, and no significant inflammatory cell infiltration.

## 4 Discussion

Infections caused by melanized fungi are increasingly recognized as a hallmark of inherited *CARD9* deficiency, reflecting the pivotal role of CARD9-mediated immunity in host defense against these opportunistic pathogens. To date, most reported cases have involved relatively well-recognized dematiaceous fungi, including *Phialophora* spp., *Exophiala* spp., *Corynespora* spp., and *Ochroconis* spp (Wang et al., 2014; Yan et al., 2016; Corvilain et al., 2018; Chen et al., 2022; Ling et al., 2022; Dan et al., 2024). In contrast, human infections caused by previously unrecognized environmental melanized fungi remain exceptionally rare. During this examination, although NGS testing of clinical sample could not be performed as corroborative evidence, repeated direct microscopy of clinical samples, culture, and re-isolation following relapse all indicated that this was a case of phaeohyphomycosis caused by an opportunistic dematiaceous fungus. Based on comparisons of ITS, LSU, TEF1-α and RPB2 sequences, as well as the ITS phylogenetic tree, we tentatively identified the organism as *Brunneofusispora hyalina*, despite the absence of more comprehensive descriptive material, such as conidiogenous structures, for morphological analysis. This finding expands the spectrum of dematiaceous fungi capable of causing disease in individuals with impaired CARD9-dependent immunity.

An increasing number of case reports and cohort studies have also highlighted a striking geographical concentration of CARD9-associated dematiaceous fungal infections in East Asia. A recent review showed that 55.17% of patients from East Asia (including China, Japan, and South Korea) were infected with dematiaceous fungi, compared with substantially lower proportions in the Middle East (10%, 1/10), North Africa (4.55%, 1/22), and Turkey (0%, 0/17). This geographical disparity has been attributed primarily to the high prevalence of the c.820dup *CARD9* founder mutation in East Asian populations (Dan et al., 2024). However, environmental exposure may also contribute (Guo et al., 2019; Latawa et al., 2022; Sarah et al., 2022; Zhang et al., 2023), as the remarkable diversity and abundance of dematiaceous fungi in East Asia, particularly in China, could increase opportunities for infection in genetically susceptible individuals. The present case further supports the concept that rare environmental fungi, previously regarded as saprotrophs, may emerge as opportunistic human pathogens when host antifungal immunity is compromised.

*B. hyalina* is a recently described melanized fungus belonging to the order *Pleosporales*. The species was first reported in 2021 as a saprotroph isolated from submerged decaying wood in a freshwater habitat in Thailand (Mark et al., 2021). Our representation expands the spectrum of pathogenic dematiaceous fungi, which are frequently implicated in phaeohyphomycosis, chromoblastomycosis, and mycetoma. These fungi are taxonomically classified primarily within the orders *Capnodiales*, *Dothideales*, *Pleosporales*, *Chaetothyriales*, *Microascales*, *Sordariales*, and *Calosphaeriales* (Revankar and Sutton, 2010; Li and Yoon, 2025). Compared to the type strain described by Calabon et al., our clinical isolate consistently failed to produce conidia despite cultivation on multiple sporulation-inducing media consistent with the absence of budding dematiaceous spores observed during direct microscopic examination of the clinical specimen. Moreover, *in vitro* culture of this isolate exhibited abundant hyphal anastomosis (Phookamsak et al., 2019; Mark et al., 2021). Hyphal anastomosis is commonly observed in mycorrhizal and phytopathogenic fungi, where it facilitates colony establishment, nutrient redistribution, and cellular communication (Glass et al., 2000; Ruiz-Roldan et al., 2010; Chagnon, 2014). Although this phenomenon has not been demonstrated in infected tissue and its role in opportunistic fungal infections remains unclear, the extensive hyphal fusion observed in culture suggests that this trait may contribute to environmental adaptation and host persistence in *B. hyalina.*

The limited thermotolerance of *B. hyalina* provides a physiological basis for several testable hypotheses rather than definitive conclusions. Because the isolate failed to grow above 35 °C and lost viability after exposure to 40 °C, it is conceivable that adjunctive local hyperthermia, such as infrared thermotherapy, could help control localized cutaneous lesions; however, this possibility has not been evaluated and should not be interpreted as a clinical recommendation. Similarly, the temperature sensitivity of the isolate is consistent with—but does not establish—a contributing role in the patient’s unusually prolonged clinical course, during which the infection remained confined to the skin for more than three decades without systemic dissemination and exhibited seasonal variation (winter exacerbation, summer partial remission). Direct evidence linking thermotolerance to either the absence of deep invasion or the seasonal pattern is lacking, and these associations remain speculative. Whole-genome analysis indicates that *B. hyalina* possesses a gene repertoire with features associated with plant-associated fungi: over 70% of the identified PHI homologs matched entries from plant pathogenic fungi. However, sequence homology to PHI-base entries does not by itself confirm a virulence function in *B. hyalina*, and a subset of these homologs correspond to deeply conserved proteins with general cellular functions rather than pathogen-specific virulence determinants. With this caveat, the observed enrichment of plant-pathogen-associated homologs is consistent with the notion that opportunistic fungi such as *B. hyalina* may rely more on host susceptibility and general fitness traits than on specialized, lineage-specific virulence factors during human infection. The relative paucity of homologs matching human-pathogen-specific virulence entries further supports this interpretation, though experimental evidence is needed to test these hypotheses directly (Gostinčar et al., 2018).

This interpretation is further supported by the murine infection model. Unlike the highly virulent dematiaceous fungi *Fonsecaea pedrosoi*, *Cladophialophora carrionii*, and *Phialophora verrucosa*, which can cause progressive disease and mortality in CARD9-deficient mice (Wu et al., 2016; Wu et al., 2018; Zhang et al., 2023), *B. hyalina* caused only transient infection in immunocompetent mice and persistent localized infection in *CARD9* knockout mice without mortality. A similar pattern of chronic infection caused by another non-sporulating dematiaceous fungus has also been reported (Guo et al., 2019). Together with the thermal growth characteristics of the isolate, these observations support a model in which the pathogenic potential of *B. hyalina* is determined by the interplay between fungal physiological constraints and host immunity. Its limited thermotolerance likely restricts deep tissue invasion and systemic dissemination, whereas impaired CARD9-mediated antifungal immunity permits persistent cutaneous colonization and chronic inflammation. *In vitro* drug susceptibility testing indicates that the new generation of triazole drugs have lower MIC values compared to fluconazole, which is consistent with previous antimicrobial results against dematiaceous fungi (Li and Yoon, 2025). In contrast, MIC of terbinafine was lower than that of the azole drugs, ranging from 0.25 to 0.5 µg/mL. Its *in vitro* potency was more than five dilution steps greater than that of amphotericin B, which is a highly encouraging finding. Further *in vitro* susceptibility testing should be conducted to establish its antifungal efficacy against dematiaceous opportunist. Building on the MIC findings, we performed infection and treatment simulation experiments in CARD9-deficient mice using voriconazole and terbinafine. The results showed decreased fungal burden and disease severity; however, complete elimination of the fungus was not achieved in CARD9-deficient mice. Considering the patient’s prolonged and recurrent illness, it can be inferred that restoring the host’s antifungal immune response is as crucial as antifungal drug treatment—a concept that requires further conclusive experimental validation. Therefore, cutaneous fungal infections caused by rare fungi in individuals with *CARD9* deficiency may not be effectively treated with antifungal medications alone but likely require long-term therapy supplemented with additional approaches such as local thermotherapy and immune-modulating strategies.

## 5 Conclusion

This study presents the first documented human case of phaeohyphomycosis caused by *Brunneofusispora hyalina* in a patient with a heterozygous *CARD9* variant, identifying this environmental fungus as a novel opportunistic pathogen. The fungus maintains its saprophytic nature in the environment and exhibits limited heat tolerance. The persistent infection and recurrence observed in this patient, coupled with antifungal treatment outcomes in *Card9* KO mice following experimental infection—which reduced fungal burden but failed to achieve complete pathogen eradication—underscore the tenacious colonization ability of this opportunist in immunocompromised hosts. These observations further highlight the formidable challenges in achieving definitive eradication of such phaeohyphomycotic infections. In light of these results, future research should prioritize investigating combined adjunctive strategies beyond conventional antifungal monotherapy to secure definitive clinical outcomes.

## Data Availability

The datasets presented in this study can be found in online repositories. The names of the repository/repositories and accession number(s) can be found in the article/**Supplementary Material**.
