# Supplementary material for: Persistent cutaneous phaeohyphomycosis caused by Brunneofusispora hyalina in a patient with a heterozygous CARD9 variant: the opportunistic potential of a newly described environmental fungus
Source: Front Fungal Biol. 2026 Oct 13;7:1938341. doi: 10.3389/ffunb.2026.1938341 (PMC13710338; doi:10.3389/ffunb.2026.1938341)
Supplement: Supplementary file 1 [file Table1.docx]

**Supplementary Materials:** Table S1: All the 54 diseases related to virulence genes; Table S2: The six pathogenicity-related genes with up than 95% identity.

**Table S1.** All the 54 diseases related to virulence genes.

| **Disease** | **Number** | **Percent** |
| --- | --- | --- |
| blast (rice) | 84 | 21.27% |
| pulmonary aspergillosis | 50 | 12.66% |
| Fusarium head blight | 47 | 11.90% |
| Fusarium ear blight | 40 | 10.13% |
| grey mold | 20 | 5.06% |
| glume blotch | 19 | 4.81% |
| brown spot | 15 | 3.80% |
| no data found | 10 | 2.53% |
| white muscardine | 9 | 2.28% |
| candidiasis | 8 | 2.03% |
| Infection | 8 | 2.03% |
| Phoma stem canker | 8 | 2.03% |
| Southern corn leaf blight | 8 | 2.03% |
| vascular wilt | 7 | 1.77% |
| aspergillosis | 5 | 1.27% |
| Fusarium wilt | 5 | 1.27% |
| Blast | 4 | 1.01% |
| leaf blight | 4 | 1.01% |
| brown spot (citrus) | 3 | 0.76% |
| ear rot (corn); yellow mold (peanut) | 2 | 0.51% |
| mycosis | 2 | 0.51% |
| root rot | 2 | 0.51% |
| Septoria nodorum blotch | 2 | 0.51% |
| spot blotch | 2 | 0.51% |
| cryptococcosis | 2 | 0.51% |
| anthracnose | 1 | 0.25% |
| anthracnose (Cucurbitaceae) | 1 | 0.25% |
| apple scab | 1 | 0.25% |
| black spot | 1 | 0.25% |
| Black spot disease | 1 | 0.25% |
| blast disease (rice) | 1 | 0.25% |
| brown spot disease | 1 | 0.25% |
| corky root rot (tomato) | 1 | 0.25% |
| denture stomatitis | 1 | 0.25% |
| glume blotch (wheat) | 1 | 0.25% |
| green muscardine | 1 | 0.25% |
| grey mould | 1 | 0.25% |
| histoplasmosis | 1 | 0.25% |
| leaf mold | 1 | 0.25% |
| leaf spot | 1 | 0.25% |
| maize anthracnose | 1 | 0.25% |
| net blotch | 1 | 0.25% |
| northern leaf blight | 1 | 0.25% |
| paracoccidioidomycosis | 1 | 0.25% |
| Septoria leaf blotch | 1 | 0.25% |
| Septoria tritici blotch | 1 | 0.25% |
| spot blotch; common root rot | 1 | 0.25% |
| Stagonospora nodorum blotch | 1 | 0.25% |
| stalk rot (corn) | 1 | 0.25% |
| stem canker | 1 | 0.25% |
| stem canker (oilseed rape) | 1 | 0.25% |
| Verticillium wilt | 1 | 0.25% |
| wheat yellow rust | 1 | 0.25% |
| White muscardine disease | 1 | 0.25% |

**Table S2.** The six pathogenicity-related genes with up than 95% identity.

| **GeneID** | **Identity%** | **ProteinID** | **PHIMolConnID** | **Gene** | **Pathogen species** |
| --- | --- | --- | --- | --- | --- |
| M648309980 | 99.5 | A0A384K6F2 | PHI:10434 | BcactA (Bcin16g02020) | *Botrytis cinerea* |
| M648309638 | 99.4 | D2X3B5 | PHI:2414 | AaGa1 | *Alternaria alternata* |
| M648301597 | 99.1 | Q96X31 | PHI:230 | PTK1 | *Pyrenophora teres* |
| M648304173 | 98.7 | P41388 | PHI:820 | beta-tubulin | *Venturia inaequalis* |
| M648313176 | 98.3 | A6RPU4 | PHI:8034 | BcATG8 | *Botrytis cinerea* |
| M648300029 | 96.9 | Q0U4L8 | PHI:7568 | HOG1 | *Parastagonospora nodorum* |
